# Occupational performance of children with autism spectrum disorder and quality of life of their mothers

**DOI:** 10.1186/s13104-021-05890-4

**Published:** 2022-01-15

**Authors:** Seyedeh Zeinab Beheshti, Seyed-Sirvan Hosseini, Saman Maroufizadeh, Amir Almasi-Hashiani

**Affiliations:** 1 School of Rehabilitation, Department of Occupational Therapy, Arak University of Medical Sciences, Arak, Iran; 2 Student Research Committee, Arak University of Medical Sciences, Arak, Iran; 3 School of Nursing and Midwifery, Guilan University of Medical Sciences, Rasht, Iran; 4 School of Health, Department of Epidemiology, Arak University of Medical Sciences, Arak, Iran; 5 Traditional and Complementary Medicine Research Center, Arak University of Medical Sciences, Arak, Iran

**Keywords:** Quality of life, Occupational performance, Autism, Autism spectrum disorder, Parents, Canadian Occupational Performance Measure

## Abstract

**Objectives:**

Limited studies were found to investigate the occupational performance of autistic children and their parents’ quality of life. Therefore, this study aimed to investigate occupational performance of children with Autism Spectrum Disorder (ASD) and QoL of their mothers.

**Results:**

In this study, 88 participants were selected from autism centers in Arak, Iran, 2020. The Canadian Occupational Performance Measure (COPM) and the parent version of Quality of Life in Autism Questionnaire (QoLA-P) were used to assess the occupational performance of ASD children and their mothers QoL. QoLA-P consists of parts A which is related to the quality of life and part-B related to the problems that these children have and are related to the parents or their caregivers. Regarding occupational performance, the first priority of mothers is self-care with frequency 64.8%. The finding suggested a significant correlation between total function score of COPM and the score of part-A (r = 0.227, p = 0.033) of QoLA-P. Also, the results revealed a significant correlation between the total satisfaction score of COPM and the score of part-A (r = 0.236, p = 0.026) and part-B of QoLA-P questionnaire (r = 0.231, p = 0.030). The mothers’ first priority is self-care and, the total satisfaction and function score of COPM showed a significant correlation with mothers’ QoL.

**Supplementary Information:**

The online version contains supplementary material available at 10.1186/s13104-021-05890-4.

## Introduction

Autism Spectrum Disorder (ASD) is a global health crisis that leads to behavioral and communication symptoms. Over the last 3 decades, the rate of autism has increased dramatically worldwide. Currently, autism affects more than 3 million people in the United States (1 case per 68 children in 2014) [1]. Epidemiological studies in Iran show a statistically growing trend of autism. The prevalence of autism in Iran in 2007 was 26.6 per 10,000 children and in 2014, 25.9 per 10,000 children [2].

Symptoms of ASD manifests itself in the form of defects in communication and social interactions and repetitive and limited patterns of behavior [3]. In addition, children with ASD experience serious problems with sensory processing, perceptual and cognitive skills, and language which can affect their occupational performance in self-care, productivity and play areas [4].

Limited occupational performance leads to limited participation. According to the International Classification Model of Function Disability and Health, participation is the involvement of the individual in life situations and is one of the important components of function and health [5]. Participation has a great impact on the growth and development of the child. With participation, the child develops the skills and competencies needed to succeed at home, community and life [6]. Studies show that children with disabilities experience more limited participation in activities than children with disabilities [7].

Child care is a main part of being a parent. Usually, many children spend their childhood with the least need for special services from health care systems. As the prevalence of developmental disorders increases, so does the parental responsibility for caring of children with special needs [8]. Providing the special needs of children with long-term functional disabilities can be very exhausting and affect the level of physical and mental health of their parents and thus lead to changes in their performance and adaptation [9].

Occupational performance problems in children with developmental disability can also affect the number, type, and quality of their caregivers activities [7]. Many studies have shown that parents of children with ASD experience higher levels of anxiety, exhaustion, isolation, sense of blame, tiredness and psychological problems than Children with other disorders [9–12]. The presence of a child with ASD in the family doesn’t only affect the patterns of parental use of time, but Grace believes that families with children with ASD aren’t satisfied with ways they participate in the activities of daily living [13].

Some studies have shown QoL of parents of autistic children is lower than of parents of typically developed children [9, 14, 15]. However, studies have been conducted on the QoL of parents of children with disabilities such as cerebral palsy [16], intellectual disability [17], down syndrome and developmental delay [18]; no study was found to investigate the occupational performance of autistic children and their parents’ QoL. Therefore, the aim of this study was to investigate occupational performance of children with ASD and QoL of their mothers.

## Main text

### Methods

#### Design and participants

In this cross-sectional study, 88participants were selected from autism school, autism rehabilitation centers and occupational therapy centers in Arak, Iran, 2020. These centers are referral centers for autistic patients in Arak, who refer to these centers for education and rehabilitation cares.

Inclusion criteria including diagnosis of autism by a neurologist and psychiatrist, ages 3 to 18, and autistic children whose main caregiver was their mothers. Mothers who did not want to continue the study and answering all the questions were excluded from the study.

After obtaining a written informed consent from the mothers, the historical and demographic information related to the child and mother was measured. Then the Canadian Occupational Performance Measure (COPM) [19] were filled through a semi-structured interview with the mothers. In addition, the parent version of Quality of Life in Autism Questionnaire (QoLA-P) [20] was also given to the mothers and they were asked to complete it.

### The Canadian Occupational Performance Measure (COPM)

COPM is a unique tool designed for occupational therapists to use over time to change the perception of the client (self-client) in their work performance and in the area of work performance and in the areas of self-care, productivity and play a game. Activities are written in the interview guide and within the appropriate COPM domain, after which the individual rates the importance of each activity on a ten-point visual analog scale, ratings range from 1 to 10. According to the manual, the participants select the five activities that are most important and ranks them based on the degree of priority. The person's self-perception of performance and satisfaction is then ranked from 1 to 10.

Dehghan et al. [21] in their study suggested that the Persian version of COPM has acceptable validity and reliability in Iranian mothers of children with cerebral palsy and in their study, the correlation coefficient of test–retest score for performance and satisfaction are reported to be 0.84 and 0.87, respectively.

### Quality of Life in Autism Questionnaire-parent version (QoLA-P)

The quality of life questionnaire for parents with ASD children has two parts, the first part (part-A) is related to the quality of life and the second part (part-B) is related to the problems that these children have and are related to the parents or their caregivers. The reliability and validity of Persian version of QoLA-P was assessed by the authors and the results showed that it is a valid questionnaire to assess the QoL of Iranian Parents of children with ASD (two subscales revealed good internal validity with Cronbach’s alpha of 0.899 and 0.950 for Part-A and Part-B, respectively).

#### Part-A (caregivers’ quality of life subscale)

The part A contains 28 items to measure parents’ overall perception of their quality of life. Each item is measured by a 5-point LIKERT scoring scale. Therefore, the scores of Section A vary from 28 to 140, higher scores indicate better QoL.

#### Part-B (difficulties children with ASD can experience)

This section is designed to examine parents' views on how autistic disorders are problematic in their lives. Here is a list of 20 items that children with ASD experience. Part B scoring is from 1 to 5. Therefore, the scores of part B vary from 20 to 100, and higher scores indicate less problems for parents with their children’s ASD-related behaviors. Based on Eapen V et al. study [20], internal consistency coefficients for the QoLA were 0.94 and 0.92 for Part A and B, respectively.

### Sample size

To determine the sample size, the amount of type 1 error was considered 5% and the study power 80%. Since no similar study was found, assuming an expected correlation coefficient of 0.3, the minimum sample size required for this study was estimated at 85 ASD children.

### Data analysis

To analyze the data, two independent T-test, likelihood ratio chi-square and correlation coefficient were used. Significance level was considered < 0.05. Version 14 of the Stata software is used for analysis of the data.

## Results

The mean age of children and their mothers were 6.43 (SD = 2.81) and 35.0 (SD = 5.3) years, respectively. Of the children with ASD, 69.3% (n = 61) were boy. Of their mothers, 44.3% were university-educated, 79.5% (n = 70) were housewife, 14.8% had unwanted pregnancy in ASD children and 61.4% (n = 54) of them gave birth by cesarean section.

As it was displayed in Table 1, regarding occupational performance, the first, second and third priority of mothers is self-care with frequency 64.8%, 43.4% and 53.4% and importance score of 9.8 (SD = 0.54), 9.5 (SD = 1.2) and 9.4 (SD = 1.3), respectively. After self-care, productivity and leisure were the most important priorities of mothers.

**Table 1:** Prioritize the occupational performance of mothers with ASD children based on main items of COPM

Mothers Priorities	1st Priority		2nd Priority		3rd Priority	
	Frequency (%)	Importance Score	Frequency (%)	Importance Score	Frequency (%)	Importance Score
Self-care	57 (64.8%)	9.8 (0.54)	47 (53.4%)	9.5 (1.2)	47 (53.4%),	9.4 (1.3)
Productivity	22 (25.0%)	9.6 (1.5)	23 (26.1%)	9.8 (0.67)	24 (27.3%),	9.2 (1.9)
Leisure	9 (10.2%)	9.7 (0.66)	18 (20.5%)	9.3 (1.4)	17 (19.3%),	9.2 (1.4)

*We assessed the occupational performance of ASD children from the perspective of their mothers

As shown in Table 2, the most important first priority of mothers’ occupational performance is related to the personal care (56.8%) with importance score of 9.8 (SD = 0.58). Following that, the play/school is mentioned as the most frequent issue (25%) with importance score of 9.6 (SD = 1.5) among the respondents as the first priority.

**Table 2:** Prioritize the occupational performance of mothers with ASD children based on sub-items of COPM

Mothers Priorities	1st Priority		2nd Priority		3rd Priority	
	Frequency (%)	Importance Score	Frequency (%)	Importance Score	Frequency (%)	Importance Score
Personal care	50 (56.8%)	9.8 (0.58)	41 (46.6%)	9.5 (1.2)	30 (34.1%)	9.4 (1.3)
Functional mobility	5 (5.7%)	10 (0)	1 (1.1%)	10 (0)	7 (7.9%)	9.7 (0.75)
Community management	2 (2.3%)	10 (0)	5 (5.7%)	9.6 (0.9)	10 (11.4%)	9.3 (1.6)
Household management	–		–		2 (2.3%)	4.5 (0.7)
Play/school	22 (25.0%)	9.6 (1.5)	23 (26.1%)	9.8 (0.67)	22 (25.0%)	9.6 (1.3)
Quiet recreation	–		5 (5.7%)	8.4 (2.1)	5 (5.7%)	9 (1.4)
Active recreation	5 (5.7%)	10 (0)	5 (5.7%)	10 (0)	7 (7.9%)	8.8 (1.8)
Socialization	4 (4.5%)	9.5 (1)	8 (9.1%)	9.5 (1.1)	5 (5.7%)	10 (0)

*We assessed the occupational performance of ASD children from the perspective of their mothers

The total function score and satisfaction score of occupational performance of mothers with ASD children are estimated 4.20 (SD = 1.98) and 3.43 (SD = 1.99), respectively. The function and satisfaction mean scores for the first three priorities are reported in Table 3. The function and satisfaction mean scores for the first priority is 4.27 (SD = 2.55) and 3.37 (SD = 2.61).

**Table 3:** The function and satisfaction mean scores for the first three priorities

Mothers priorities	1st Priority	2nd Priority	3rd Priority
Function	4.27 (2.55)	4.37 (2.49)	3.98 (2.52)
Satisfaction	3.37 (2.61)	3.62 (2.49)	3.11 (2.56)

*We assessed the occupational performance of ASD children from the perspective of their mothers

The correlation of total function score and satisfaction score of occupational performance of mothers with ASD children and the scores of part A and B of QoLA-P questionnaire are depicted in Additional file 1: Fig S1–Additional file 4: Fig S4. The finding suggested a significant correlation between total function score of COPM and the score of part-A (r = 0.227, p = 0.033) (Additional file 1: Fig S1) of the quality of life questionnaire for parents, while its correlation with the score of part-B was not statistically significant (r = 0.129, p = 0.230) (Additional file 2: Fig S2). Also, regarding the total satisfaction score of COPM, a significant correlation was observed between this score and the score of part-A (r = 0.236, p = 0.026) (Additional file 3: Fig S3) and part-B of QoLA-P questionnaire (r = 0.231, p = 0.030) (Additional file 4: Fig S4).

## Discussion

The main results of this study are that in terms of occupational performance in children with ASD, the three mothers’ first priority is self-care followed by productivity and leisure. Furthermore, the total satisfaction score and function score of COPM showed a significant correlation with mothers’ QoL score.

According to the results, self-care is the first problem of children from the perspective of most parents. This area of occupation is so important that it is mentioned as the second and third problems. The area of self-care in this study includes personal care, mobility and social management. Personal care activities have been the most important in the first three priorities of mothers, which shows that the mothers of children participating in this study seek to improve self-care skills in their children. Personal care activities are the main self-care tasks that people usually do daily and include eating, sleeping, bathing, toileting, dressing, hygiene and mobility, and can be a challenge to children with ASD [22, 23]. Our results are consistent with previous studies [7, 22–25].

However, different studies show different results in determining the educational and rehabilitation priorities of children with ASD. By study of 22 families Rodgers reported that communication skills were the main priority for families to educate and rehabilitate their children [26]. In the studies of Whitaker and Spann, social and communication growth were also identified as first priorities [27, 28].

The next priority that parents of autistic children focused on was productivity and play/school activities. Given the age of the children studied, who were 3 to 18 years old, it is expected that play and school activities as the one of main occupation of these children will be considered by parents. Play/school in the productivity area include, group plays, playing with toys and doing homework. Communication problems in children with ASD severely limit group play in these children [29]. The limited interests and tastes of these children can also affect the way and variety of toys they use [30]. Ghanizadeh states that children with autism are less attractive to their peers, which may be due to problems in social interactions or repetitive behaviors [31].

In examining the quality of life of parents and its relationship with occupational performance of their children, it was found that there is a significant positive relationship between part-A of QoL-A questionnaire with total function score COPM and both part-A and B with total satisfaction score. Functional problems in children with developmental disorders lead to the need for care services that go far beyond the normal needs of children and the expectations of their parents [9].

## Conclusion

The three mothers' first priority is self-care followed by productivity and leisure. Furthermore, the total satisfaction score and function score of COPM showed a significant correlation with mothers’ QoL score.

## Limitations and recommendations

One of the limitations of this study is relatively limited sample size, which may affect the results. In addition, another limitation of this study was that the children’s work performance questionnaire was completed by mothers, which can also affect the results.

Therefore, it is recommended that similar studies with larger sample sizes be designed to achieve more valid results. It is also recommended that similar studies be performed on children with high-functioning autism.

## Supplementary Information

**Additional file 1: Figure S1.** The correlation of total function score of COPM and QoLA-P (part A).

**Additional file 2: Figure S2.** The correlation of total function score of COPM and QoLA-P (part B).

**Additional file 3: Figure S3.** The correlation of total satisfaction score of COPM and QoLA-P (part A).

**Additional file 4: Figure S4.** The correlation of total satisfaction score of COPM and QoLA-P (part B).

## Data Availability

The datasets used and/or analyzed during the current study are available from the corresponding author on reasonable request.

## Abbreviations

ASD: Autism spectrum disorder
QoL: Quality of life
QoLA: Quality of Life in Autism Questionnaire
COPM: The Canadian Occupational Performance Measure
QoLA-P: The parent version of Quality of Life in Autism Questionnaire
S.D.: Standard deviation
