# Supplementary figures and images for: Occupational performance of children with autism spectrum disorder and quality of life of their mothers
Source: BMC Res Notes. 2022 Jan 15;15:18. doi: 10.1186/s13104-021-05890-4 (PMC8760686; doi:10.1186/s13104-021-05890-4)

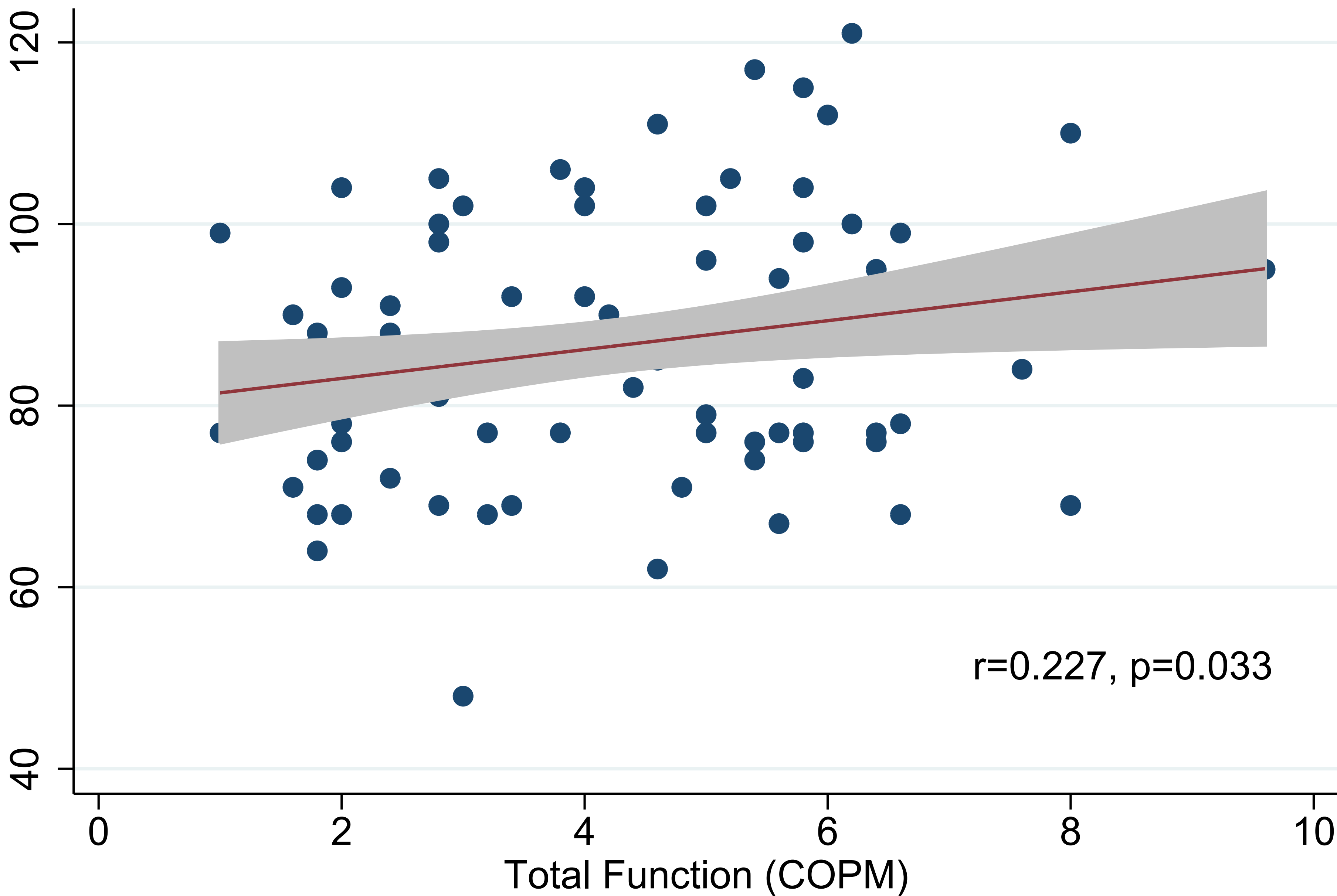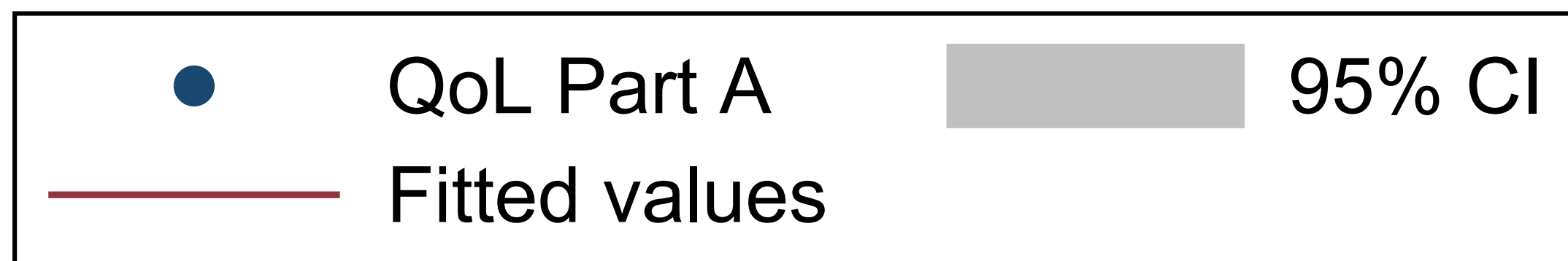

Supplement: Supplementary file 1 — Additional file 1: Figure S1. The correlation of total function score of COPM and QoLA-P (part A). [file 13104_2021_5890_MOESM1_ESM.pdf]

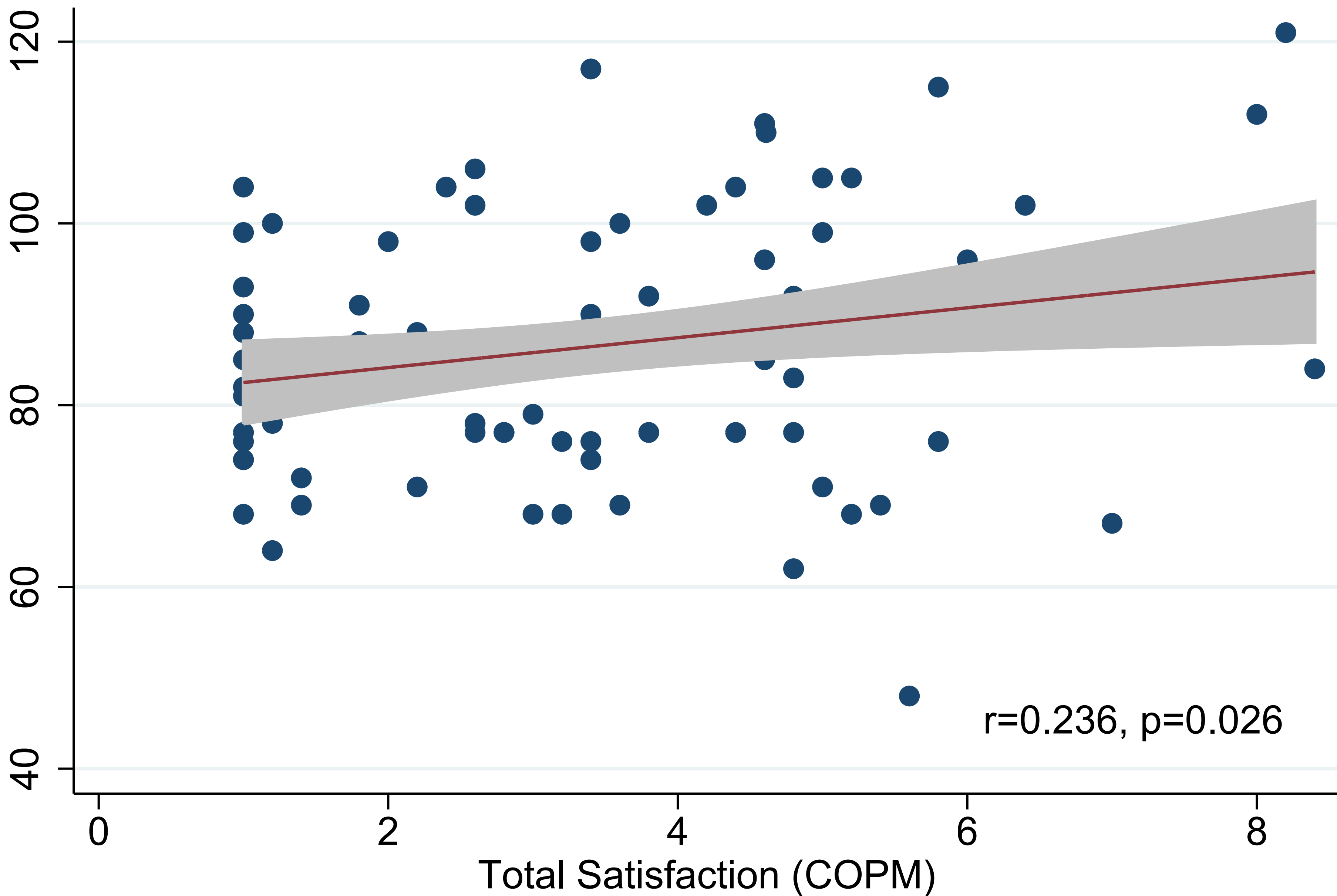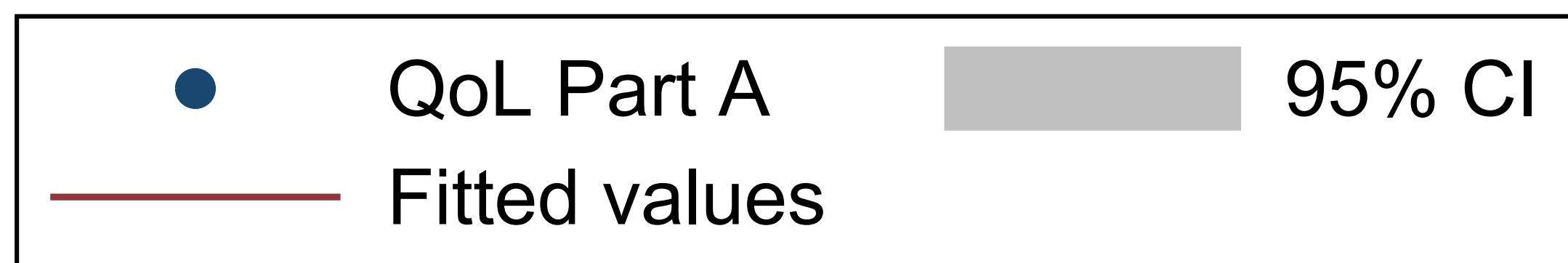

Supplement: Supplementary file 2 — Additional file 2: Figure S2. The correlation of total function score of COPM and QoLA-P (part B). [file 13104_2021_5890_MOESM2_ESM.pdf]

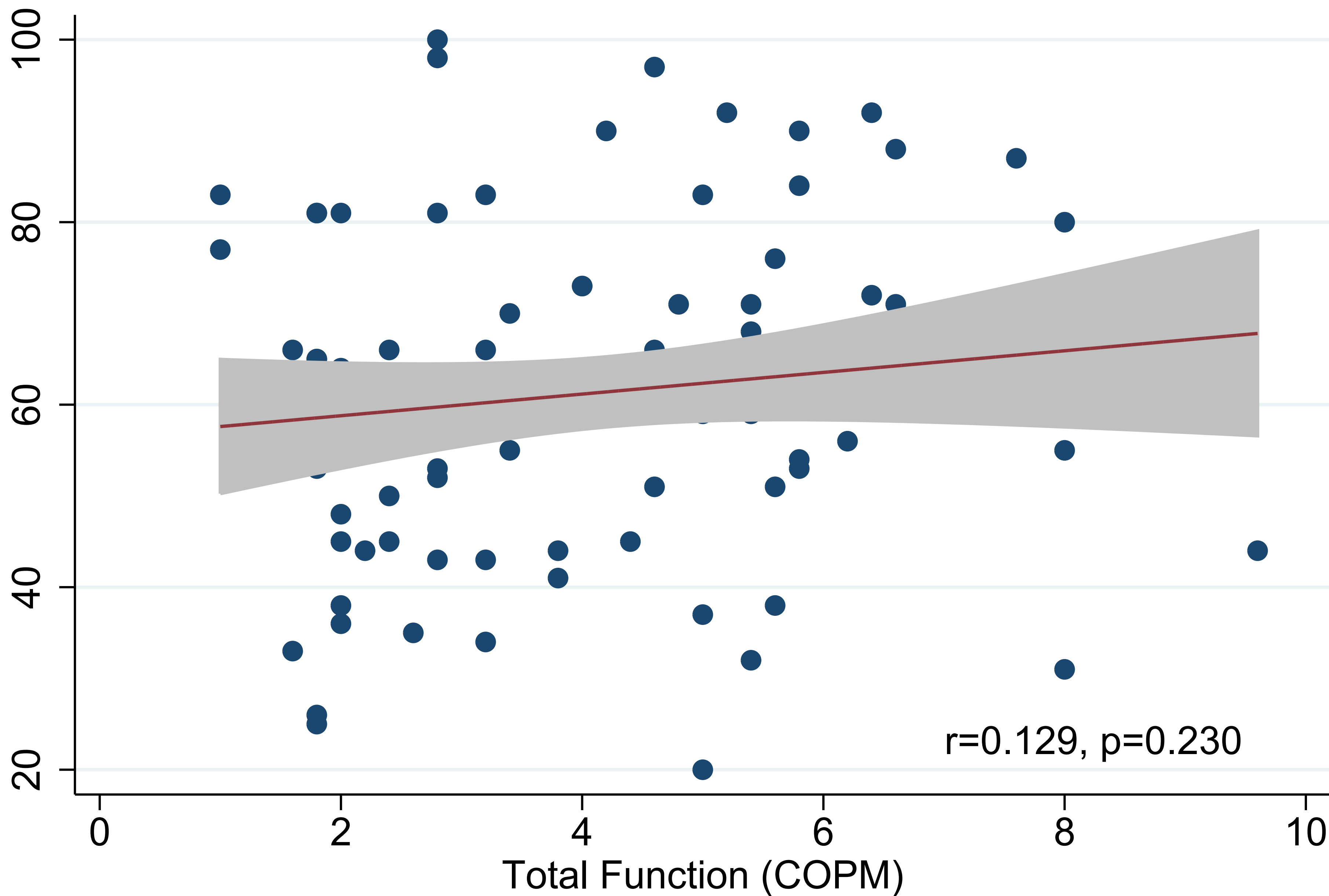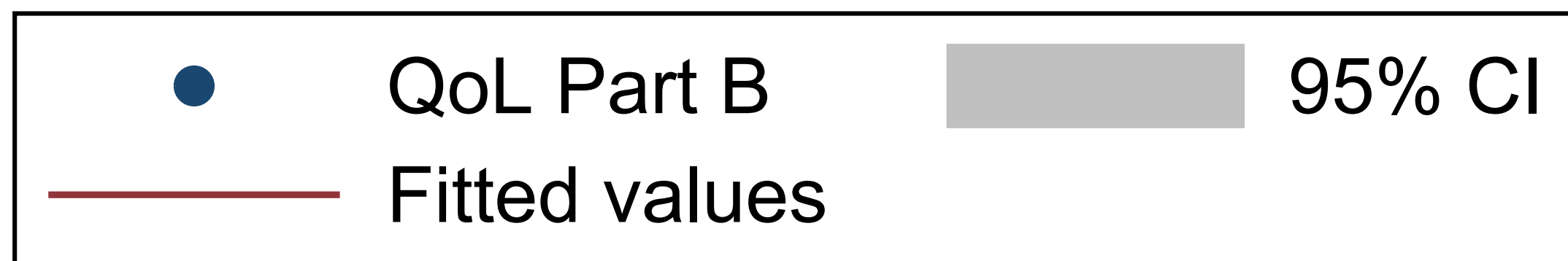

Supplement: Supplementary file 3 — Additional file 3: Figure S3. The correlation of total satisfaction score of COPM and QoLA-P (part A). [file 13104_2021_5890_MOESM3_ESM.pdf]

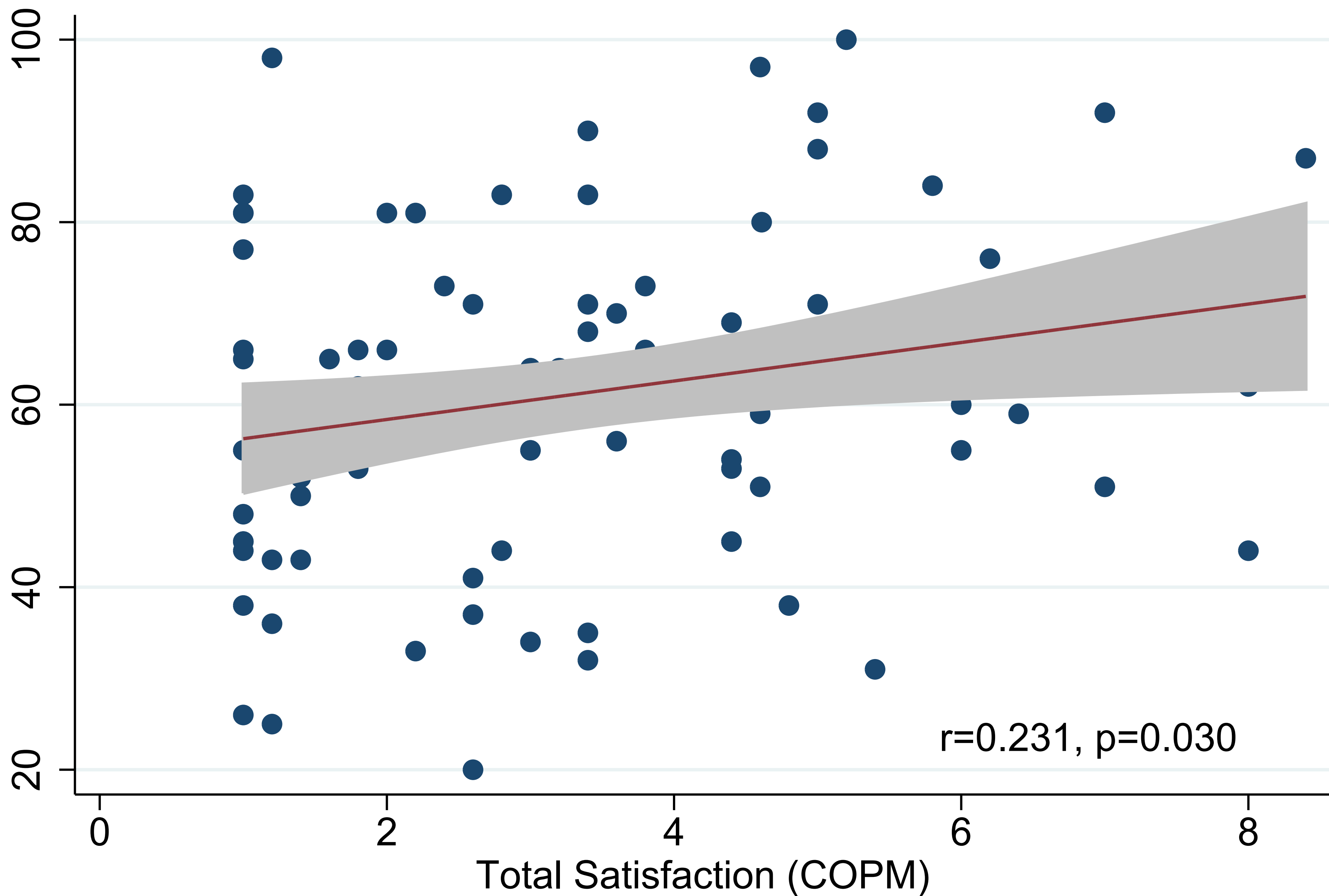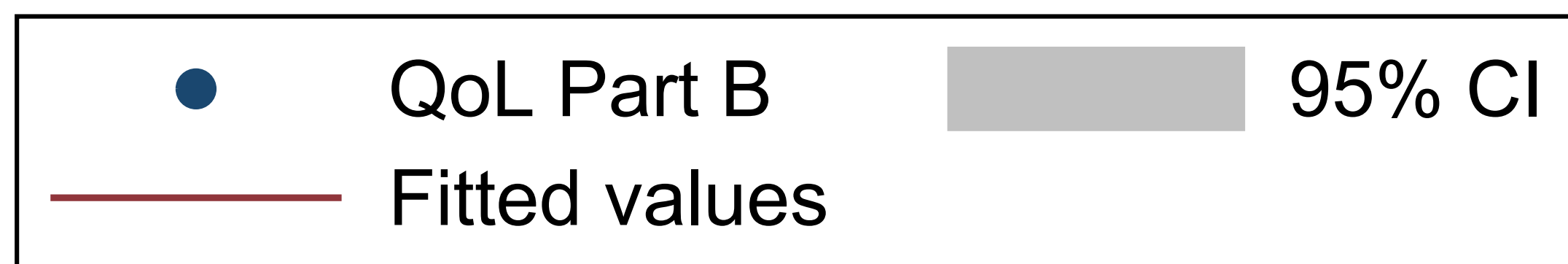

Supplement: Supplementary file 4 — Additional file 4: Figure S4. The correlation of total satisfaction score of COPM and QoLA-P (part B). [file 13104_2021_5890_MOESM4_ESM.pdf]
